# Author Correction: A high-throughput neutralizing antibody assay for COVID-19 diagnosis and vaccine evaluation

**DOI:** 10.1038/s41467-021-24287-2

**Published:** 2021-06-22

**Authors:** Antonio E. Muruato, Camila R. Fontes-Garfias, Ping Ren, Mariano A. Garcia-Blanco, Vineet D. Menachery, Xuping Xie, Pei-Yong Shi

**Affiliations:** 1 Department of Biochemistry and Molecular Biology, University of Texas Medical Branch, Galveston, TX USA; 2 Department of Microbiology and Immunology, University of Texas Medical Branch, Galveston, TX USA; 3 Department of Pathology, University of Texas Medical Branch, Galveston, TX USA; 4 Programme in Emerging Infectious Diseases, Duke-NUS Medical School, Singapore, Singapore; 5 Department of Internal Medicine, University of Texas Medical Branch, Galveston, TX USA; 6 Institute for Human Infections and Immunity, University of Texas Medical Branch, Galveston, TX USA; 7 Sealy Institute for Vaccine Sciences, University of Texas Medical Branch, Galveston, TX USA; 8 Sealy Center for Structural Biology & Molecular Biophysics, University of Texas Medical Branch, Galveston, TX USA; 9 Institute for Translational Science, University of Texas Medical Branch, Galveston, TX USA

**Keywords:** Infectious-disease diagnostics, SARS-CoV-2

Correction to: *Nature Communications* 10.1038/s41467-020-17892-0, published online 13 August 2020.

The original version of this Article contained an error in the Acknowledgements, which incorrectly listed the NIH grant ‘AI142759’. This has been corrected in both the PDF and HTML versions of the Article.

